# Editorial: Oxidative Stress Revisited—Major Role in Vascular Diseases, Volume II

**DOI:** 10.3389/fphys.2021.826129

**Published:** 2022-01-12

**Authors:** Cristina M. Sena, Raquel Seiça, George Perry

**Affiliations:** ^1^Institute of Physiology, Faculty of Medicine, University of Coimbra, Coimbra, Portugal; ^2^Department of Neuroscience, Developmental and Regenerative Biology, University of Texas at San Antonio, San Antonio, TX, United States

**Keywords:** oxidative stress, inflammation, vascular, non-communicable diseases, novel therapeutic approaches

Oxidative stress has been described as an imbalance between oxidants and antioxidants with a disruption of redox signaling and control (Harman, 1956; Sies, 1985). Our understanding of oxidative stress has evolved to one of homeostatic balance, where stress is met with increased defenses and novel and chronic balances involving hundreds of subsystems underlying aging and chronic disease. It is now well-established that reactive oxygen species (ROS) are pleiotropic signaling molecules that play an important role in redox signaling in physiological oxidative eustress (i.e., low-level physiological oxidative stress). Supra-physiological levels of ROS cause tissue damage to proteins, lipids and DNA and are defined as oxidative distress (Sies, 2020). Dissection of these complex systems reveals new therapeutic avenues. Oxidative stress is an underlying cause of many non-communicable diseases such as cardiovascular and neurodegenerative diseases, affecting different organs of our organism (Figure 1).

**Figure 1:**
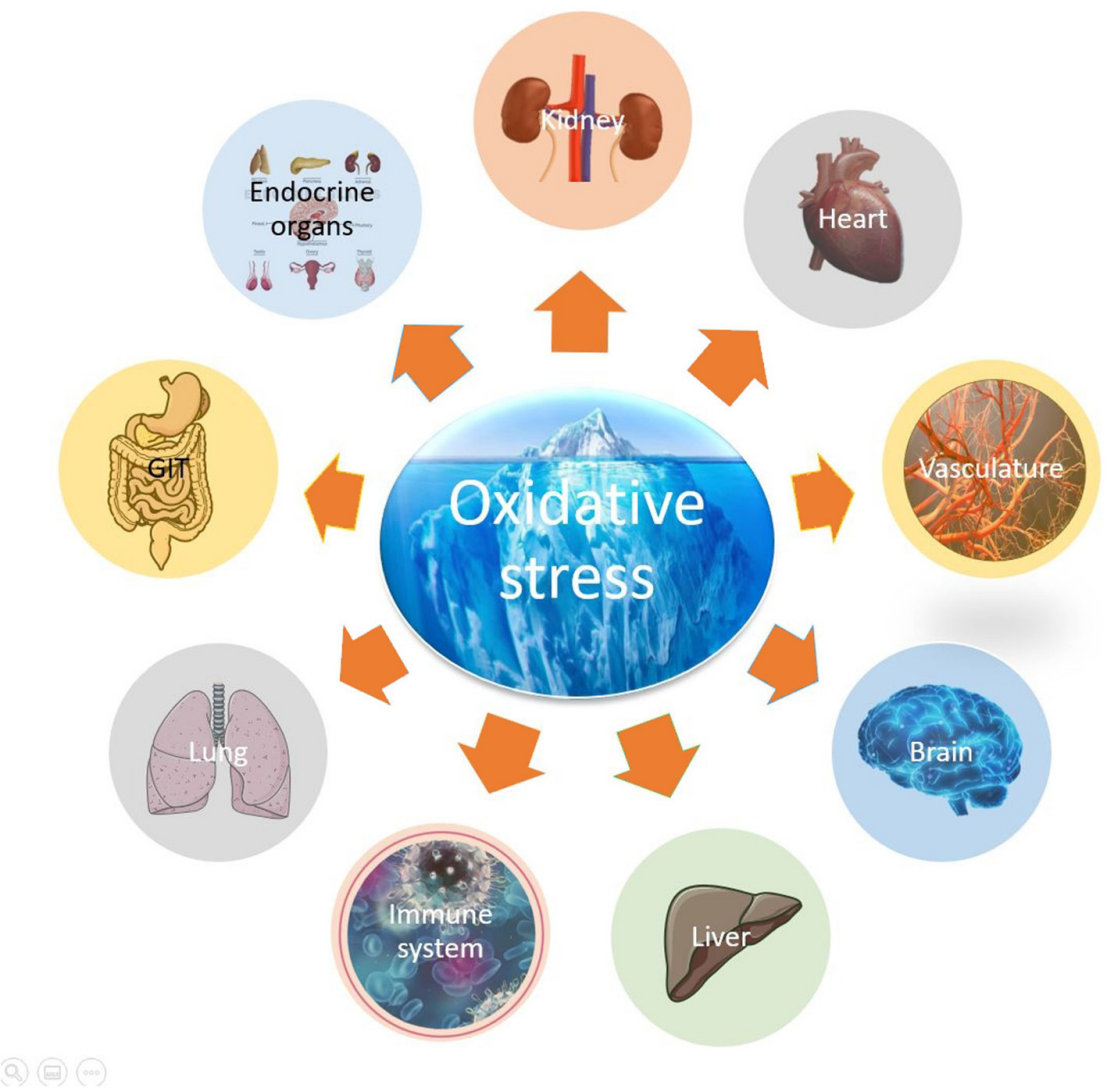
Oxidative stress as an underlying cause of multiple organ damage.

In vascular disease, oxidative stress promotes endothelial dysfunction and inflammation, affecting numerous cells in the vascular wall (Sena et al., 2019). This Research Topic focuses on oxidative stress in vascular pathophysiology and highlights different strategies to inhibit ROS production. While many studies focused on neutralization of ROS (superoxide anion, peroxynitrite, and hydrogen peroxide) others try to reduce the vicious circle associated with oxidative stress.

In this Research Topic a review of the relationship between cerebral small vessel disease, oligodendrocyte dysfunction and sleep disorders focusing in oxidative stress as a common event and its possible role in the onset of Alzheimer's disease was performed by Lloret et al. In addition, Lorenço and Laranjinha reviewed the glutamate-NMDA receptor-nNOS axis in neurovascular (NVC) coupling and the role of oxidative stress. Some therapeutical strategies targeting the rescue or maintenance of nitric oxide bioavailability with potential to mitigate the NVC dysfunction associated with neurodegenerative conditions were also discussed.

Xia et al. performed an oral treatment of apolipoprotein E-knockout mice with the sirtuin 1 activator resveratrol that led to a reduction of nicotinamide adenine dinucleotide phosphate oxidase activity in the heart. This was associated with reduced membrane translocation of the small GTPase Rac1 and p67phox.

A revision of the molecular alterations in smooth muscle cells of pulmonary artery under hypobaric hypoxia conditions, including oxidative stress, inflammation, kinase activation and signaling, nitric oxide and HIF pathways, among others was performed by Siques et al.

This Frontiers Research Topic in oxidative stress is a view of the more recent research in the field pointing to some gaps in knowledge and news avenues to understand the role of oxidative stress in non-communicable diseases.

## Author Contributions

CS drafted the editorial. RS and GP read and modified the editorial. All authors contributed to the article and approved the submitted version.

## Funding

This study was supported by National Funds *via* FCT (Foundation for Science and Technology) through the Strategic Project UIDB/04539/2020 and UIDP/04539/2020 (CIBB).

## Conflict of Interest

The authors declare that the research was conducted in the absence of any commercial or financial relationships that could be construed as a potential conflict of interest.

## Publisher's Note

All claims expressed in this article are solely those of the authors and do not necessarily represent those of their affiliated organizations, or those of the publisher, the editors and the reviewers. Any product that may be evaluated in this article, or claim that may be made by its manufacturer, is not guaranteed or endorsed by the publisher.
